# DNA methylation levels are associated with CRF_1_ receptor antagonist treatment outcome in women with post-traumatic stress disorder

**DOI:** 10.1186/s13148-018-0569-x

**Published:** 2018-11-03

**Authors:** Julius C. Pape, Tania Carrillo-Roa, Barbara O. Rothbaum, Charles B. Nemeroff, Darina Czamara, Anthony S. Zannas, Dan Iosifescu, Sanjay J. Mathew, Thomas C. Neylan, Helen S. Mayberg, Boadie W. Dunlop, Elisabeth B. Binder

**Affiliations:** 1 Department of Translational Research in Psychiatry, Max Planck Institute of Psychiatry, Munich, Germany; 2 Department of Psychiatry and Behavioral Sciences, Emory University School of Medicine, Atlanta, GA USA; 3 Department of Psychiatry and Behavioral Sciences, University of Miami Miller School of Medicine, Miami, FL USA; 4 Department of Psychiatry, Icahn School of Medicine at Mount Sinai, New York, NY USA; 5 Menninger Department of Psychiatry & Behavioral Sciences, Baylor College of Medicine & Michael E. Debakey VA Medical Center, Houston, TX USA; 6 Department of Psychiatry, University of California, San Francisco, San Francisco, CA USA; 7 The San Francisco Veterans Affairs Medical Center, San Francisco, CA USA; 8 Department of Psychiatry and Behavioral Sciences, Duke University Medical Center, Durham, NC USA; 9 New York University School of Medicine, New York, NY USA; 10 Nathan Kline Institute for Psychiatric Research, Orangeburg, NY USA

**Keywords:** CRF_1_ receptor antagonist, DNA methylation, Epigenetics, PTSD, CRHR1, NR3C1, FKBP5

## Abstract

**Background:**

We have previously evaluated the efficacy of the CRF_1_ receptor antagonist GSK561679 in female PTSD patients. While GSK561679 was not superior to placebo overall, it was associated with a significantly stronger symptom reduction in a subset of patients with probable CRF system hyperactivity, i.e., patients with child abuse and *CRHR1* SNP rs110402 GG carriers. Here, we test whether blood-based DNA methylation levels within *CRHR1* and other PTSD-relevant genes would be associated with treatment outcome, either overall or in the high CRF activity subgroup.

**Results:**

Therefore, we measured *CRHR1* genotypes as well as baseline and post-treatment DNA methylation from the peripheral blood in the same cohort of PTSD-diagnosed women treated with GSK561679 (*N* = 43) or placebo (*N* = 45). In the same patients, we assessed DNA methylation at the PTSD-relevant genes *NR3C1* and *FKBP5*, shown to predict or associate with PTSD treatment outcome after psychotherapy. We observed significant differences in *CRHR1* methylation after GSK561679 treatment in the subgroup of patients with high CRF activity. Furthermore, *NR3C1* baseline methylation significantly interacted with child abuse to predict PTSD symptom change following GSK561679 treatment.

**Conclusions:**

Our results support a possible role of *CRHR1* methylation levels as an epigenetic marker to track response to CRF_1_ antagonist treatment in biologically relevant subgroups. Moreover, pre-treatment *NR3C1* methylation levels may serve as a potential marker to predict PTSD treatment outcome, independent of the type of therapy. However, to establish clinical relevance of these markers, our findings require replication and validation in larger studies.

**Trial registration:**

NCT01018992. Registered 6 November 2009.

**Electronic supplementary material:**

The online version of this article (10.1186/s13148-018-0569-x) contains supplementary material, which is available to authorized users.

## Background

Post-traumatic stress disorder (PTSD) is a common psychiatric disorder with a prevalence of about 5% in the general population and an overall lifetime prevalence of 7–12%. Key symptoms of the disorder include intrusive memories, avoidance, and numbing as well as hyperarousal. Typically, these symptoms are long lasting and occur after exposure to traumatic life events. Women are twice as likely to develop the disease than men. PTSD therapies include both evidence-based psychotherapies and pharmacology, but only few patients attain remission. Currently, only two medications, paroxetine and sertraline, are approved by the US Food and Drug Administration (FDA). These SSRIs are capable of significantly reducing PTSD symptoms, but with only 20–30% remission rates to these agents, there is a need for additional pharmacologic treatment options [1].

Among pathophysiologic mechanisms that have been investigated for PTSD, disruptions of regulation of the hypothalamic-pituitary-adrenal (HPA) axis are among the most frequently cited hypotheses [2]. A key regulator of the HPA axis is the corticotropin-releasing factor (CRF) and its type 1 receptor (CRF_1_ receptor), and many studies have reported alterations in this system in PTSD [3]. Therefore, it represents a promising novel drug target for this disorder. In response to stress, CRF is secreted by nerve terminals of the paraventricular nucleus of the hypothalamus and binds to the CRF_1_ receptor in the adenohypophysis to release adrenocorticotropic hormone (ACTH). This process acts as the initial step of HPA axis activation and leads to the release of a number of hormones from the adrenal cortex including cortisol. Numerous studies in laboratory animals as well as in humans indicate that abnormalities of these HPA axis regulators play a crucial role in stress-related disorders such as PTSD [4].

In humans, for example, a number of independent studies report increased cerebrospinal fluid concentrations of corticotropin-releasing factor in PTSD patients [5–7], suggesting hyperactivity of the hypothalamus and extra-hypothalamus CRF system. Moreover, previous investigations have found that genetic variants in the CRF receptor 1 gene (*CRHR1*) are associated with differences in CRF signaling and may also impact individual responses to environmental stressors [3]. The most studied are variants within a haplotype tagged by the intronic SNP rs110402 that also comprises rs242924 and rs7209436. Interactions with exposure to child abuse and this haplotype were shown to alter risk for major depression, with individuals homozygous for the G-allele of rs110402 and exposed to child abuse being at higher risk in several but not all studies (see [8] for review). This haplotype has also been associated with differences in the neural activation profile with emotional stimulus processing [9], as well as neuroendocrine responses in psychological and pharmacological challenge tests [10–14], in which individuals who experienced childhood abuse and carry the G-allele display stronger HPA axis disturbances.

These preclinical and clinical results, taken together, support the role of CRF/CRF_1_ receptor as a potential drug target in PTSD. However, antagonism of the CRF_1_ receptor may only benefit those patients with initial increases in CRF signaling, which according to the above cited endocrine studies are likely to be those with exposure to child abuse and carrying the G-allele of rs110402.

We recently published a study evaluating the efficacy of a novel CRF_1_ receptor antagonist (GSK561679) in a cohort of female PTSD patients in a double-blind, placebo-controlled trial. Although the drug was not superior to placebo overall, it was associated with a significantly stronger symptom reduction in a subset of patients with probable CRF_1_ receptor hyperactivity, i.e., patients with childhood abuse and carriers of the GG genotype of the *CRHR1* SNP rs110402 [15, 16]. These patients may represent a biologically distinct subtype of PTSD and show distinct biomarker profiles. Markers that predict or monitor treatment outcome would represent an important tool to offer targeted treatment for individual patients. Despite great progress in identifying the underpinnings of the pathophysiology of PTSD and some very promising results in the biomarker field [17, 18], there is still no clinically applicable marker in PTSD, neither for diagnosis nor, perhaps even more significantly, to guide treatment selection. This is likely due to the complex pathophysiology of the disease that may include an interplay of genetics, environment, and epigenetic changes. It is therefore likely that not a single but rather a combination of different biological and clinical markers will need to be identified [18].

In addition to gene variants that predispose to PTSD development, epigenetic changes have been implicated in the pathophysiology of PTSD (for review, see [19]). These modifications may also serve as diagnostic marks as well as predicting and monitoring treatment outcome. Several studies highlight the possible use of epigenetic marks in peripheral tissues such as the blood and saliva as diagnostic markers in PTSD [18, 20, 21]. So far, epigenetic marks of only two genes, also within the HPA axis, *NR3C1*—encoding the glucocorticoid receptor (GR) and *FKBP5*—a co-chaperone of the GR, have been shown to associate with treatment response. More specifically, *NR3C1* baseline promoter methylation in peripheral blood predicted treatment outcome in PTSD, and in the same study, promoter methylation of *FKBP5* decreased in association with symptom improvement [22]. These findings were observed after 12 weeks of psychotherapy and have not yet been investigated in the context of pharmacological treatment.

Extending our previous study showing potential effects of a novel CRF_1_ receptor antagonist (GSK561679) in a specific subset of women with PTSD (GG homozygous for rs110402 and with a history of childhood abuse) [16], we here use the same cohort to test whether blood-based epigenetic changes of PTSD relevant genes could serve as potential markers for treatment selection and outcome monitoring in biologically defined subgroups of patients. Given that the drug targets the CRF_1_ receptor, we focused our analysis on the methylation of the *CRHR1* gene using the previous subgrouping of patients based on genetic and environmental risk factors. In addition, we explored whether methylation levels of two other genes within the stress hormone system (*NR3C1* and *FKBP5*), previously shown to predict and correlate with PTSD symptom improvement after psychotherapy [22], would also be associated with pharmacological treatment response in our study, again with specific focus on patients with probable CRF system hyperactivity (rs110402 GG-carriers and exposure to child abuse).

## Results

### Subgroup differences in *CRHR1* baseline methylation and change in *CRHR1* methylation from baseline to post-treatment

**Fig. 1:**
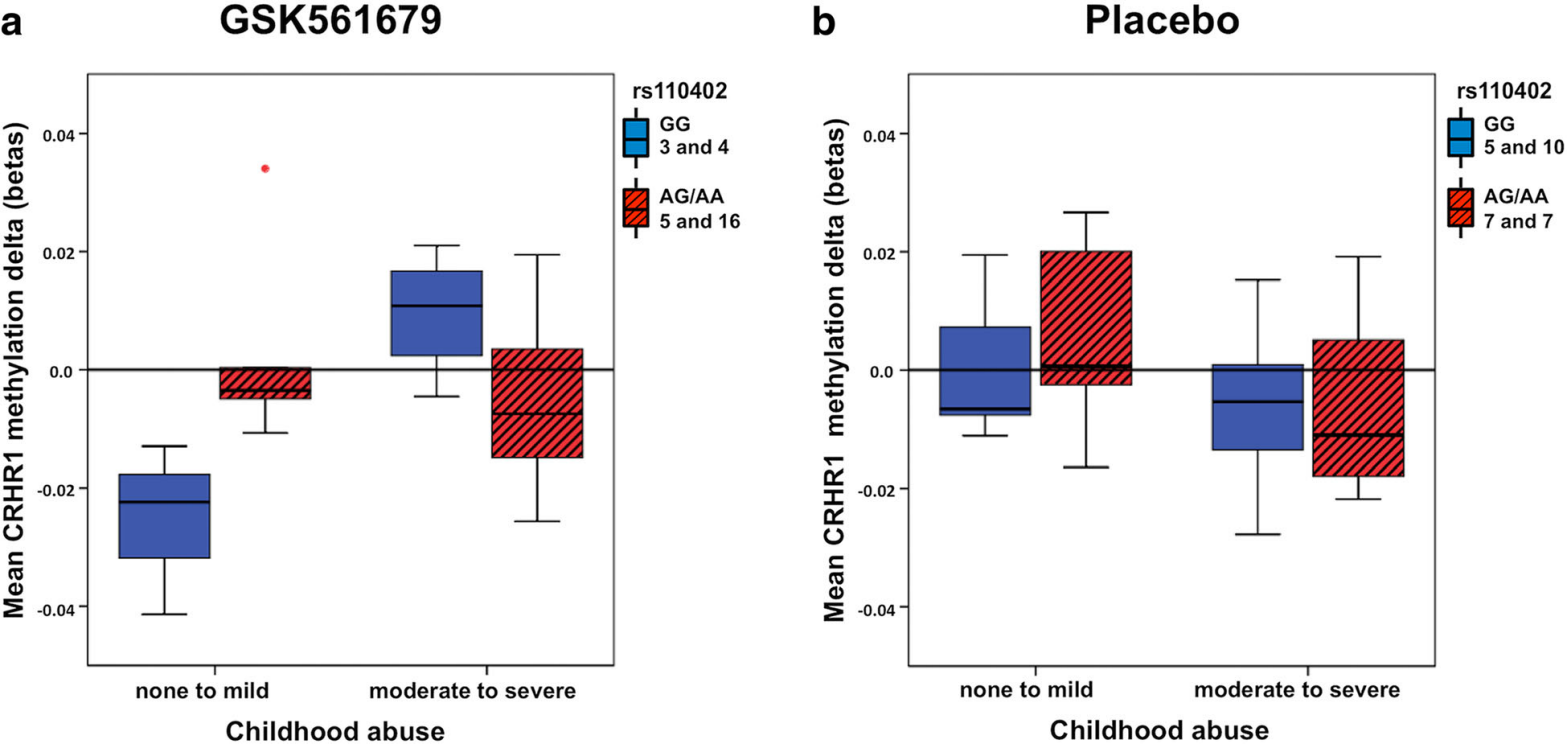
The boxplots describe the mean change of CRHR1 methylation (top tertile of the most variable CpGs from pre- to post-treatment) in abused and non-abused patients treated with GSK561679 or placebo. GG carriers are shown in blue (plain boxes) and AA/AG in red (striped boxes). Positive values correspond to an increase, whereas negative values correspond to a decrease in methylation from baseline to endpoint. Dots indicate outliers. Three-way interaction of treatment × rs110402 A carrier status × child abuse was significantly associated with mean methylation change (*n* = 57; *p* = 0.033) (**a**, **b**). After treatment stratification, there was a significant interaction effect of rs110402 A carrier status and child abuse on mean methylation change in subjects treated with GSK561679 (*n* = 28; *p* = 0.00005) (**a**) but not with placebo (*n* = 29; *p* > 0.05) (**b**)

First, we tested a model with the main effects and interaction effect of child abuse and rs110402 carrier status on mean *CRHR1* baseline methylation. Seventy-nine subjects were included in this analysis due to missing genotype data in three samples. Neither the main effects nor the interaction effect showed significance (*n* = 79; *p* > 0.05). Next, we tested a model including main effects of treatment as well as interaction effects of treatment by child abuse, treatment by rs110402, child abuse by rs110402, and the three-way interaction of treatment by child abuse by rs110402 on changes in mean methylation levels of *CRHR1* from baseline to post-treatment. Due to missing methylation data in two baseline samples and one post-treatment sample, 57 subjects with baseline and post-treatment methylation data remained for this analysis. There was a significant interaction effect of child abuse by rs110402 carrier status (*n* = 57; *F* (1, 41) = 9.05; *p* = 0.004; *ß* = − 0.449; Cohen’s *f* = 0.47; *R*^2^ = 0.38; adj. *R*^2^ = 0.153; post-hoc power = 0.94) on change in methylation. Further, the three-way interaction of treatment by child abuse by rs110402 showed a significant effect on *CRHR1* methylation levels from pre- to post-treatment (*n* = 57; *F* (1, 41) = 4.86; *p* = 0.033; *ß* = − 0.297; Cohen’s *f* = 0.344; *R*^2^ = 0.38; adj. *R*^2^ = 0.153; post-hoc power = 0.72) (Fig. 1a, b).

### Genotype by childhood abuse interaction on methylation change stratified by treatment

To further explore the significant three-way interaction on *CRHR1* methylation, we investigated the interaction of rs110402 carrier status by child abuse on the change in methylation levels stratified by treatment. The interaction showed a significant effect on pre- to post-treatment *CRHR1* methylation change only in patients treated with the CRF_1_ receptor antagonist (*n* = 28; *F* (1, 16) = 29.81; *p* = 0.00005; withstands Bonferroni correction for multiple testing; *ß* = − 0.913; Cohen’s *f* = 1.366; *R*^2^ = 0.73; adj. *R*^2^ = 0.55; post-hoc power = 0.99) (Fig. 1a).

Interestingly, the subset of patients with child abuse and who are also carriers of the GG genotype of rs110402 showed an increase in *CRHR1* methylation with GSK561679 treatment. This subgroup was previously described to benefit most from the drug ([16] and Additional file 1: Figure S1). The other three subsets of patients (no abuse and rs110402 GG; no abuse and rs110402 AG/AA; abuse and rs110402 AG/AA) showed no change or decreased methylation after GSK561679 treatment. There was no significant effect in the placebo group (*n* = 29; *p* > 0.05) (Fig. 1b).

### Baseline methylation by treatment interaction effects on PTSD symptom change

We next tested whether baseline methylation predicted %-change of PTSD symptoms from pre- to post-treatment. Seventy-nine (CAPS)/78 (PSS) subjects were included in the analysis due to missing genotype data in three samples and missing phenotype data (PSS %-change) in one sample. Neither *NR3C1* (*n* = 79/78; *p* > 0.05) nor *FKBP5* (*n* = 79/78; *p* > 0.05) showed a significant interaction effect of treatment by baseline methylation on symptom change.

### Three-way interaction effects on PTSD symptom change with treatment, baseline methylation, and SNP/child abuse

Next, we included either rs110402 or child abuse in our analysis and tested for two three-way interaction effects (rs110402 × treatment × mean baseline methylation or child abuse × treatment × mean baseline methylation) on symptom reduction measured by change in Clinician-Administered PTSD Scale (CAPS) and PTSD Symptom Scale-Self-Report (PSS-SR) scores. Treatment by baseline methylation by rs110402 carrier status was not significantly associated with differences in PTSD symptom change for neither of the genes (NR3C1: *n* = 79/78, *p* > 0.05; FKBP5: *n* = 79/78, *p* > 0.05).

The three-way interaction that included child abuse was significant for *NR3C1* baseline methylation (*n* = 78; *F* (1, 56) = 4.26; *p* = 0.044; *ß* = 0.276; Cohen’s *f* = 0.277; *R*^2^ = 0.33; adj. *R*^2^ = 0.087; post-hoc power = 0.67) and showed a trend towards significance for *FKBP5* baseline methylation (*n* = 79, *F* (1, 57) = 2.81; *p* = 0.099; *ß* = 0.215; Cohen’s *f* = 0.222; *R*^2^ = 0.28; adj. *R*^2^ = 0.017;post-hoc power = 0.38).

**Fig. 2:**
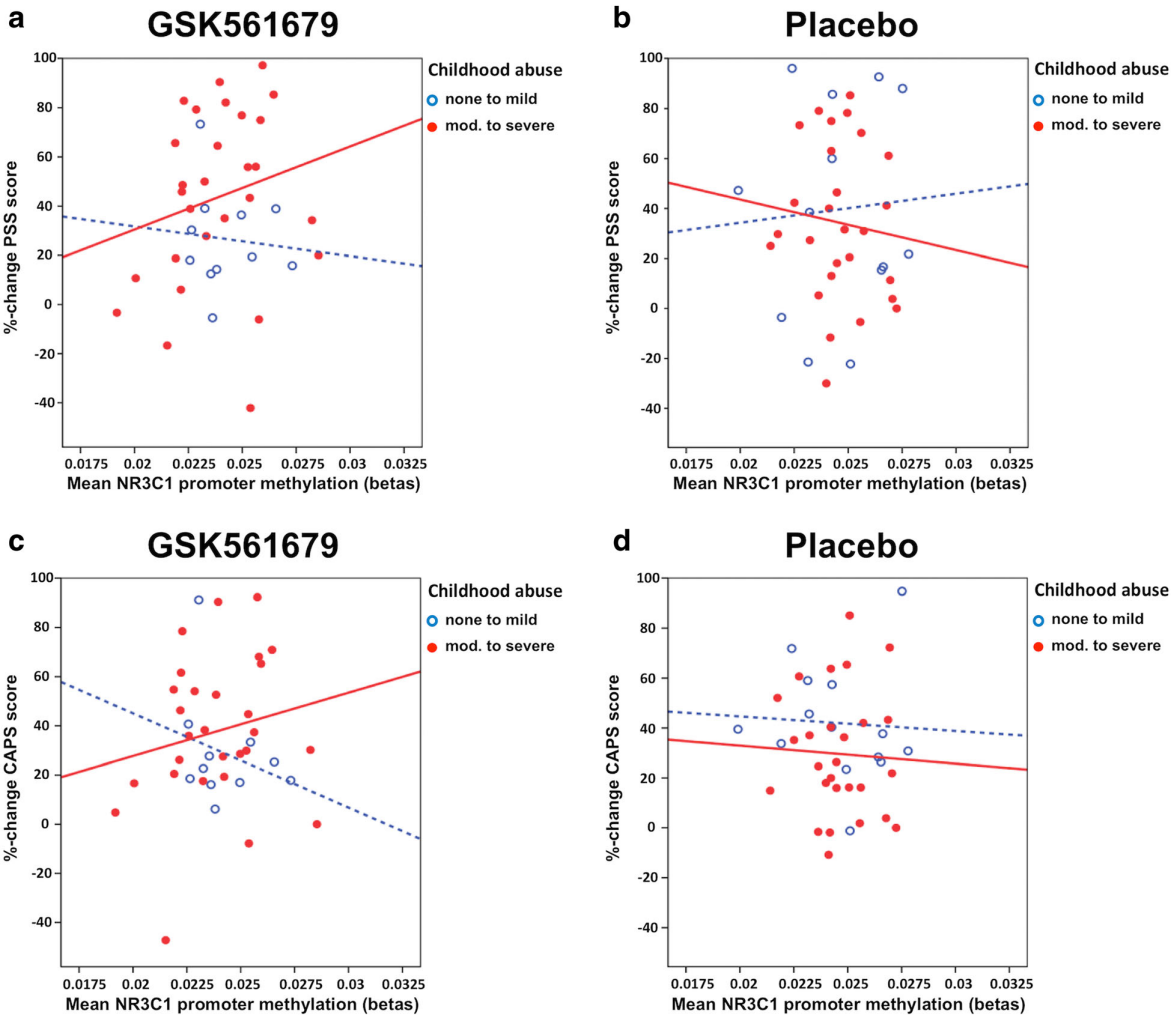
The scatter plots describe the association between the mean percent change of PTSD symptoms and mean NR3C1 methylation dependent on child abuse in patients treated with GSK561679 (**a**, **c**) or placebo (**b**, **d**). Higher symptom percent change corresponds to improvement (reduction) in PTSD symptoms from baseline to endpoint. Abused patients are shown in red (solid line) and non-abused patients in blue (dashed line). Three-way interaction of NR3C1 baseline methylation × treatment × child abuse was significantly associated with PSS %-change (*n* = 79; *p* = 0.044) (**a**, **b**) but not with CAPS %-change (*n* = 78; *p* > 0.05) (**c**, **d**). After treatment stratification, there was a significant interaction effect of baseline methylation and child abuse on PSS %-change in subjects treated with GSK561679 (*n* = 38; *p* = 0.045) (**a**) but not with placebo (*n* = 40; *p* > 0.05) (**b**). For CAPS %-change, the effect pointed in the same direction without reaching significance (**c**, **d**)

More specifically, CRF_1_ receptor antagonist-treated, abused patients with high baseline *NR3C1* methylation levels showed the strongest PSS percent change and therefore the best treatment outcome overall (Fig. 2a, b). A post-hoc analysis revealed that the interaction of baseline *NR3C1* methylation and child abuse was significantly associated with PSS percent change after CRF_1_ receptor antagonist treatment (*n* = 38; *F* (1, 20) = 4.58; *p* = 0.045; *ß* = 0.331; Cohen’s *f* = 0.478; *R*^2^ = 0.67; adj. *R*^2^ = 0.39; post-hoc power= 0.81) (Fig. 2a) but not placebo (*n* = 40; *p* > 0.05) (Fig. 2b). Results from the same analysis using CAPS score %-change as treatment outcome showed the same direction of effects but did not reach significance (three-way interaction: *n* = 79; *p* > 0.05) (Fig. 2c, d).

**Fig. 3:**
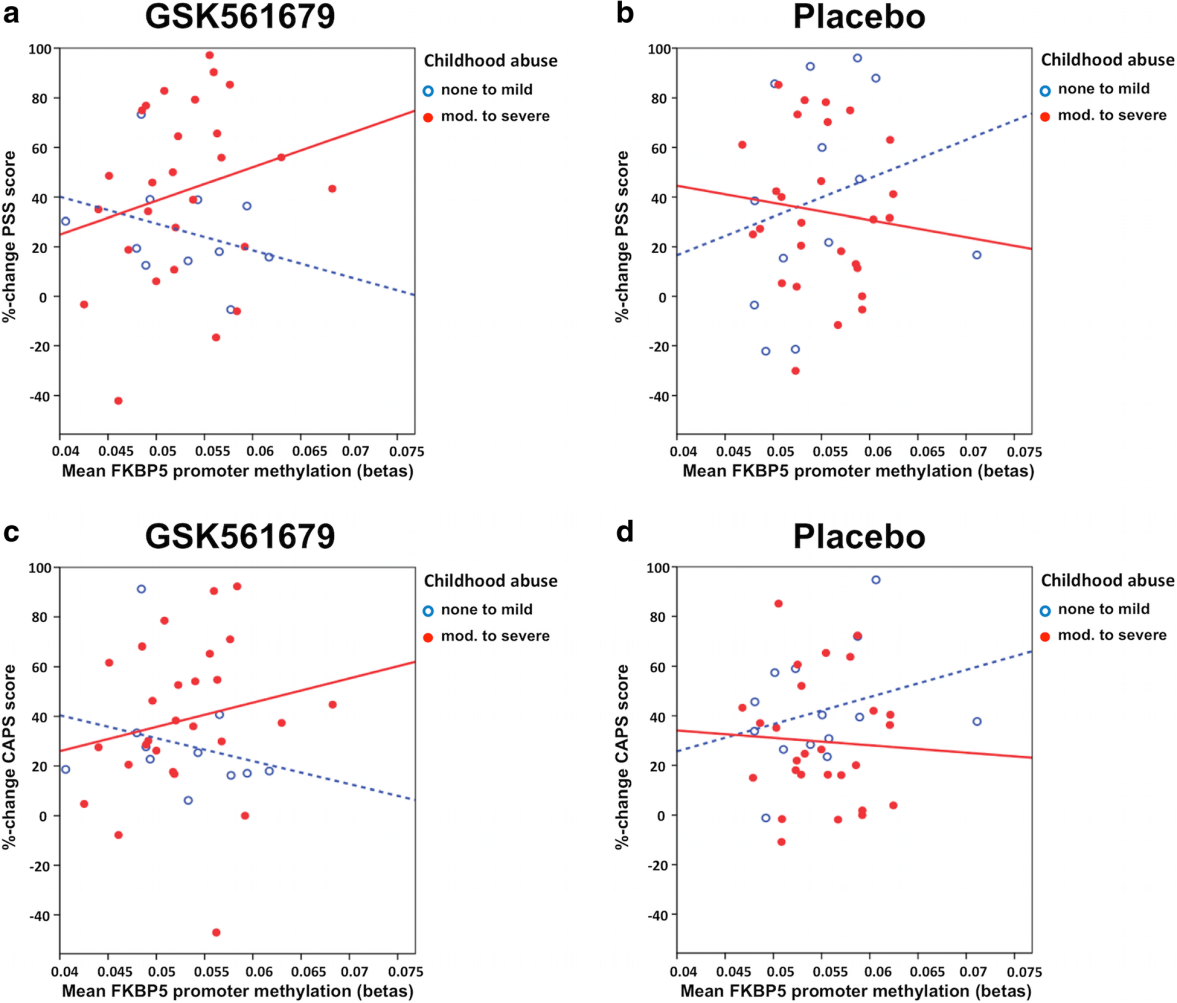
The scatter plots describe the association between the mean percent change of PTSD symptoms and mean FKBP5 methylation dependent on child abuse in patients treated with GSK561679 (**a**, **c**) or placebo (**b**, **d**). Higher symptom percent change corresponds to improvement (reduction) in PTSD symptoms from baseline to endpoint. Abused patients are shown in red (solid line) and non-abused patients in blue (dashed line). The three-way interaction testing FKBP5 baseline methylation × treatment × child abuse on CAPS %-change had a *p* value of *p* = 0.099 with an *n* = 79 (**c**, **d**) and *p* > 0.05 with PSS %-change (*n* = 78) (**a**, **b**). After treatment stratification, there was no significant interaction effect of baseline methylation by child abuse on PTSD symptom %-change in neither one of the treatment groups (*p* > 0.05 for all) (**a**–**d**)

For FKBP5, abused patients with high baseline methylation and treated with the CRF_1_ receptor antagonist experienced the strongest CAPS percent change (*n* = 79, *F* (1, 57) = 2.81; *p* = 0.099). The post-hoc analysis, stratifying patients by treatment and testing the interaction effect of baseline methylation by child abuse on PTSD symptom change, did not reach significance in neither one of the treatment groups (*p* > 0.05 for all) (Fig. 3a–d).

### Pre- to post-treatment methylation change by treatment interaction effects and three-way interaction effects including SNP or child abuse on PTSD symptom change

To examine the association between FKBP5/NR3C1 methylation change from baseline to post-treatment and symptom improvement, we tested for interaction effects of treatment by pre- to post-methylation change on %-change of PTSD symptoms from pre- to post-treatment. For NR3C1 and FKBP5, 57 subjects were included in the analysis due to missing methylation data in two baseline samples and one post-treatment sample. None of the tested interactions reached significance (FKBP5: *n* = 57, *p* > 0.5; NR3C1: *n* = 57, *p* > 0.5). Further, including either rs110402 or child abuse in our analysis to test for two three-way interactions (rs110402 × treatment × pre- to post-methylation change or child abuse × treatment × pre- to post-methylation change) on symptom reduction also did not show significant effects (FKBP5: *n* = 57, *p* > 0.5; NR3C1: *n* = 57, *p* > 0.5).

## Discussion

The objective of this study was to investigate epigenetic marks of PTSD-related genes in association with PTSD symptom changes after CRF_1_ receptor antagonist (GSK561679) treatment in female PTSD patients. In a first analysis, we observed significant differences in *CRHR1* methylation levels after treatment among patients with probable CRF hyperactivity who previously demonstrated the greatest clinical benefit from the CRF_1_ receptor antagonist [16]; this effect was not present among those who received placebo. This subgroup of patients who had experienced child abuse and were homozygous for the rs110402 GG allele were the only individuals showing a significant increase in *CRHR1* methylation from baseline to the post-GSK561679 treatment time point. All other subjects either showed no change or a reduction in methylation over the time of treatment. On the other hand, baseline *CRHR1* methylation did not predict treatment outcome, suggesting that this epigenetic change may only serve as a potential tracker of symptom changes. The maximum difference in mean *CRHR1* methylation between the subgroups was more than 3%, a change comparable to or even larger than other studies examining peripheral blood DNA methylation and psychiatric disorders or psychiatric treatment response. In fact, when examining the 11 CpGs composing the *CRHR1* variable methylation score, the maximal effects were observed in CpGs cg27410679 and cg04194664. In the subgroup of patients with child abuse and homozygous for the rs110402 GG allele, these CpGs showed an increase in methylation of up to 3.9% and a maximum methylation difference between the four subgroups of 9.9% (cg04194664) and 7.7% (cg27410679). Future studies should evaluate these optimized markers in larger samples.

A number of factors can contribute to changes in DNA methylation. In a mixed tissue such as peripheral blood, the most likely contributor is the changes in immune cell subtype composition. Changes in immune responses have been reported in PTSD (reviewed by [23]), and symptom normalization may be associated with a change in immune function and cell type proportion [24–26]. We attempted to account for this using a bioinformatics deconvolution method for blood cell types from genome-wide methylation data [27] and adding the estimated cell type proportions as covariates. In addition, there has been increasing evidence suggesting that dynamic methylation changes, as observed in our study, may be mediated by certain transcription factors [28–30]. Several studies have reported on the potential role of the glucocorticoid receptor as one of these transcription factors mediating glucocorticoid-induced DNA demethylation [31, 32]. CRF_1_ receptor antagonists influence the regulation of the HPA axis and by that, ultimately, modulate GR activity. Our previously identified subgroup of patients with rs110402 GG genotype and a history of child abuse displayed a significant increase in *CRHR1* methylation after GSK561679 treatment. Previous studies have shown that this combination of environmental and genetic risk is associated with specific disruptions of HPA axis regulation, including an enhanced cortisol response to the Trier Social Stress Test and the combined dexamethasone suppression/CRF stimulation test [11–14]. A combination of increased CRF activity and GR activation may exist in this subgroup and normalize with specific CRF_1_ receptor antagonist treatment. In fact, a number of studies have also reported GR supersensitivity with PTSD [33, 34] and its normalization with effective treatment [35, 36]. Such a reversal of GR supersensitivity in the subset of patients with response to the antagonist may also lead to changes in GR-mediated DNA methylation. In fact, active GR response elements are shown in the ENCODE project for the *CRHR1* locus [37]. Finally, GSK561679 itself could directly impact *CRHR1* methylation. However, the *CRHR1* expression is low in peripheral blood cells (https://gtexportal.org/), suggesting that the epigenetic regulation of the locus indirectly via receptor blockade and adaptive transcriptional regulation is an unlikely mechanism for inducing this effect.

In our second analysis, we investigated peripheral blood DNA methylation of two genes, for which a previous study had found an association with improvement of PTSD symptoms after prolonged exposure therapy [22]. In a small cohort of combat veterans diagnosed with PTSD, the authors reported that pre-treatment *NR3C1* methylation significantly predicted treatment outcome, with higher *NR3C1* methylation at baseline associated with better response to psychotherapy. The authors also observed a decrease in *FKBP5* promoter methylation over treatment in patients showing clinical improvement [22].

Similar to Yehuda et al. [22], we also find that higher baseline methylation of *NR3C1* is associated with better treatment outcome with the antagonist. However, in our analysis, this is only seen in patients who had also experienced child abuse. No association was found for *FKBP5*, neither for baseline levels predicting treatment outcome nor for change in *FKBP5* methylation being associated with symptom improvement, as reported in Yehuda et al. [22]. While exploratory, our results support the conclusion that peripheral blood DNA methylation of *NR3C1* is associated with PTSD treatment response.

The major limitation of this study is the small sample size, particularly after biological subgrouping. Power calculation for our main hypothesis (change of CRHR1 methylation over treatment and prediction of treatment outcome), however, revealed that power would be sufficient to detect medium to large effect sizes, whereas smaller effect sizes would have been missed. A post-hoc power analysis for the specific effect sizes detected in our study showed that power ranged between 0.673 and 0.999. Further, due to the exploratory nature of our study, we did not apply a systematic correction for multiple testing, increasing the risk for false-positive associations. To identify smaller effects, confirm our results, and reduce the risk of a type I and type II error, much larger sample sizes will be required for future studies.

An additional limitation to this study, which represents a general issue in DNA methylation analyses of mixed tissues, is to rule out cell type composition variation as a potential confounding factor contributing to the observed epigenetic changes. As described, we applied a commonly used bioinformatics cell-type deconvolution method [27] to address this issue. However, this method only accounts for six different cell types in the blood, so that changes in subtypes not covered by this algorithm may still contribute to the observed changes in DNA methylation.

## Conclusion

Overall, our results indicate that markers for PTSD likely will need to be an index, comprised of several combination markers. Here, we describe the association of *CRHR1* DNA methylation with treatment response, but only in a specific subset of patients defined by genetic and environmental risk factors. While our association of baseline *NR3C1* methylation with PTSD treatment outcome is supportive of previous findings, both studies are small. Given the exploratory nature of the study and the small sample size, larger studies that stratify patients by potential biomarker status will be needed to fully establish the clinical value of these measures.

## Methods

### Study overview

Detailed descriptions of the trial design and the study results were published previously [15, 16] and are summarized in the following.

### Cohort

Patients were recruited at four academic sites (Emory University, Icahn School of Medicine at Mount Sinai, Baylor College of Medicine, University of California San Francisco/San Francisco Veterans Affairs Medical Center) in the USA. The institutional review boards at each study site approved the study. The cohort used for this study consisted of 88 female patients between 18 and 65 years of age. Males were excluded due to potential reproductive organ toxicity of the investigational medication. All subjects were free of psychotropic medication (except non-benzodiazepine hypnotics) for at least 2 weeks prior to randomization. Subjects had to fulfill criteria for a primary psychiatric diagnosis of DSM-IV-defined PTSD of at least 3 month’s duration since the index trauma. PTSD status at the baseline (randomization) visit had to be of at least moderate severity, defined as Clinician-Administered PTSD Scale (CAPS) for DSM-IV [38] past-month and past-week total scores ≥ 50. Important exclusion criteria included current or past diagnosis of a psychotic disorder, bipolar disorder, or obsessive-compulsive disorder. Subjects with a positive test for drugs of abuse at the screening visit, or who met criteria for substance abuse or dependence within 3 months of the randomization visit, or who presented with significant current suicidal ideation were excluded. Pregnant or lactating women and subjects with an unstable medical condition were also excluded.

### Study design

Subjects participated in a parallel-group, double-blind, placebo-controlled randomized clinical trial of a novel CRF_1_ receptor antagonist (GSK561679). After randomization, patients were either treated with a nightly dose of 350 mg GSK561679 or placebo over 6 weeks. At the baseline visit (prior to treatment phase), numerous data including demographics, vital signs, and several psychiatric measures were assessed, e.g., level of childhood maltreatment was tested using the Childhood Trauma Questionnaire (CTQ). CAPS score and PTSD Symptom Scale-Self-Report (PSS-SR) [39] were assessed at weeks 1, 2, 4, and 6 after randomization to assess PTSD symptom severity, and the percent change of these scores from pre- to post-treatment were used to determine the degree of improvement in PTSD symptoms. For biological assessments (e.g., methylation levels, genotyping), whole blood was collected at baseline (*n* = 88) as well as after 5 weeks of treatment (*n* = 60 with both baseline and post-treatment) and DNA extraction was performed.

### DNA extraction

DNA isolation from whole blood was performed with a *magnetic bead*-based technology on the chemagic 360 extraction robot using the chemagic DNA Blood Kit special (PerkinElmer Inc., Waltham, MA, USA). Quality and quantity of the extracted DNA were assessed using the Epoch Microplate Spectrophotometer (BioTek, Winooski, VT, USA).

### Genotyping

Genome-wide SNP genotyping was performed for all subjects using Illumina HumanOmniExpress-24 BeadChips according to the manufacturer’s protocol. We excluded the relatives of individual subjects from the whole sample (*n* = 3, Pihat ≥ 0.0625) based on mean identity by descent (IBD) in PLINK [40]. Eighty-five subjects remained for further QC. For the genome-wide analyses that were used to correct for population stratification, we only included individuals with a sample-wise call rate ≥ 0.98 and SNPs with call rate ≥ 0.98, Hardy Weinberg equilibrium test (HWE) *p* value ≥ 1 × 10^− 5^ and MAF ≥ 0.05, allowing for a total of 575,455 markers in 85 individuals. To correct for population stratification in an ethnically mixed sample, principal components (PC) for the genetic background were calculated from all genotypes for each of the individuals using genome-wide complex trait analysis (GCTA) [41].

### Methylation analysis

DNA methylation levels were assessed using the Illumina 450k array. After bisulfite conversion with the Zymo EZ-96 DNA Methylation Kit (Zymo Research, Irvine, CA. USA), genome-wide DNA methylation levels were assessed for 84 baseline samples and 60 matching post-treatment samples using Illumina 450K DNA methylation arrays (Illumina, San Diego, CA, USA) as previously published [42].

#### Quality control of DNA methylation

Minfi Bioconductor R package (version 1.10.2) was used to perform quality control of methylation data including normalization, intensity readouts, cell type composition estimation, and beta and *M* value calculation. A detection *p* value larger than 0.01 in at least 75% of the samples led to an exclusion of the probe. Probes that were located close (10 bp from query site) to a SNP which had a minor allele frequency of ≥ 0.05 in any of the populations represented in the sample were removed as well as X chromosome, Y chromosome, and non-specific binding probes. The data were then normalized using functional normalization, which is an extension of quantile normalization included in the minfi R package. The Bioconductor R package shinyMethyl version 0.99.3 was used to identify batch effects by inspecting the association of the first principal component of the methylation levels with plate, sentrix array, and position using linear regression and visual inspection of PCA plots. A linear regression model was fitted in R with the *M* values for each probe as the dependent variable and plate, sentrix array, and row as the independent variables as factors to remove batch effects. Two baseline samples and one post-treatment sample did not pass quality control, which resulted in 82 baseline samples and 57 matching pairs with 450K methylation data.

### Statistical analyses

Statistical analysis was carried out using SPSS v.18.0 (IBM Corp., Armonk, NY, USA) and R software v 3.2 (https://www.r-project.org/). Genotype analysis (SNP rs110402): the intronic SNP rs110402 has been shown to be associated with HPA axis hyperactivity [11, 14, 43]. This may result in a different response to antagonizing the CRF system, depending on a patient’s rs110402 genotype. We therefore focused on rs110402 genotype stratification in our analysis. Direct genotypes were taken from the HumanOmniExpress-24 array (rs110402 MAF = 0.401, HWE test *p* value = 0.52). According to our previous study [16], patients were categorized by rs110402 A-allele carrier status (GG = 33 carriers and 53 A-allele carriers, of which 38 patients had the AG genotype and 15 were homozygous for the A-allele). Grouping individuals carrying one or two copies of the minor A-allele of rs110402 has been used in previous studies [9, 11, 44] and helps to preserve power. Additive effects of that SNP have previously been reported [45]. Methylation analysis: *CRHR1*: From the *CRHR1* gene locus covered by 33 CpGs on the 450k array, the top tertile (11 CpGs) of the CpGs with the most variable methylation change from pre to post-treatment was selected (Additional file 1: Table S1). The mean methylation of these 11 CpGs was calculated and used for further analysis. *NR3C1*: Mean methylation of 5 CpG sites within the 1F promoter and exon present on the Illumina 450K array was used for the analysis (Additional file 1: Table S2). DNA methylation in the 1F promoter and exon had been shown to predict PTSD treatment outcome [22]. *FKBP5*: Mean methylation level of 3 CpG sites within the exon 1 promoter present on the Illumina 450K array was used for the analysis (Additional file 1: Table S3). DNA methylation of this locus was shown to track with symptom improvement [22]. Childhood trauma status was defined as previously described by categorizing individuals as having experienced either no or only mild abuse versus those having experienced at least one type of moderate to severe abuse (emotional abuse ≥ 13, physical abuse ≥ 10, sexual abuse ≥ 8) (57 = abused, 31 = non-abused) using the CTQ [45]. We performed linear regression models adjusted for age, smoking, ancestry PC, and estimated blood cell count to test for main/two-way and three-way interaction effects on methylation changes as well as main/two-way and three-way interactions effects on PTSD symptom %-change. For each of the analysis, only individuals with complete phenotype, methylation data, genotypes, and any additional covariates were included in the model. We calculated power post-hoc using G Power 3.1 [46]. Alpha was set to 0.05, and the number of groups, degrees of freedom, and eta squares were set according to the test-specific calculations performed in SPSS. Statistical significance was considered at *p* < 0.05. Due to the exploratory nature of the study, no correction for multiple testing was applied. As a measure of effect size, Cohen’s *f* was calculated and interpreted as follows: *f* < 0.25 = small effect size; 0.25 < *f* < 0.4 = medium effect size; *f* > 0.4 = large effect size [47].

## Additional file

Additional file 1: **Figure S1.** The boxplots describe the mean % change of PSS total score in abused and non-abused patients treated with the CRHR1 antagonist or placebo. GG carriers are shown in blue (plain boxes) and AA/AG in red (striped boxes). rs110402 A carrier status by childhood abuse exposure showed a significant interaction effect on PSS score % change over treatment in subjects treated with the CRHR1 antagonist (*n* = 43; *F* (1, 31) = 4.42; *p* = 0.043) (a) but not in subjects treated with placebo (*n* = 42, *p* > 0.05) (b). rs110402 GG carriers exposed to child abuse displayed the highest % change of PSS symptoms following CRHR1 treatment. (From Biological Psychiatry; Dunlop et al., 2017). **Table S1.** CRHR1: List of CpGs used for analysis. **Table S2.** NR3C1: List of CpGs used for analysis. **Table S3.** FKBP5: List of CpGs used for analysis. (DOC 977 kb)

## Abbreviations

ACTH: Adrenocorticotropic hormone
CAPS: Clinician-Administered PTSD Scale
CpG: Cytosine-phosphate-guanine
CRF1: Corticotropin-releasing hormone receptor 1
*CRHR1*: Corticotropin-releasing hormone receptor 1 (gene)
CTQ: Childhood Trauma Questionnaire
*FKBP5*: FK506-binding protein 51 kDa gene
GR: Glucocorticoid receptor
HPA: Hypothalamic-pituitary-adrenal
*NR3C1*: Nuclear receptor subfamily 3 group C member 1
PSS: PTSD symptom scale
PTSD: Post-traumatic stress disorder
QC: Quality control
*SKA2*: Spindle and kinetochore-associated complex subunit 2
SNP: Single nucleotide polymorphism
