# Supplementary material for: DNA methylation levels are associated with CRF1 receptor antagonist treatment outcome in women with post-traumatic stress disorder
Source: Clin Epigenetics. 2018 Nov 3;10:136. doi: 10.1186/s13148-018-0569-x (PMC6215613; doi:10.1186/s13148-018-0569-x)
Supplement: Supplementary file 1 — Figure S1. The boxplots describe the mean % change of PSS total score in abused and non-abused patients treated with the CRHR1 antagonist or placebo. GG carriers are shown in blue (plain boxes) and AA/AG in red (striped boxes). rs110402 A carrier status by childhood abuse exposure showed a significant interaction effect on PSS score % change over treatment in subjects treated with the CRHR1 antagonist (n = 43; F (1, 31) = 4.42; p = 0.043) (a) but not in subjects treated with placebo (n = 42, p > 0.05) (b). rs110402 GG carriers exposed to child abuse displayed the highest % change of PSS symptoms following CRHR1 treatment. (From Biological Psychiatry; Dunlop et al., 2017). Table S1. CRHR1: List of CpGs used for analysis. Table S2. NR3C1: List of CpGs used for analysis. Table S3. FKBP5: List of CpGs used for analysis. (DOC 977 kb) [file 13148_2018_569_MOESM1_ESM.doc]

**Additional file 1**

**Figure S1**

**
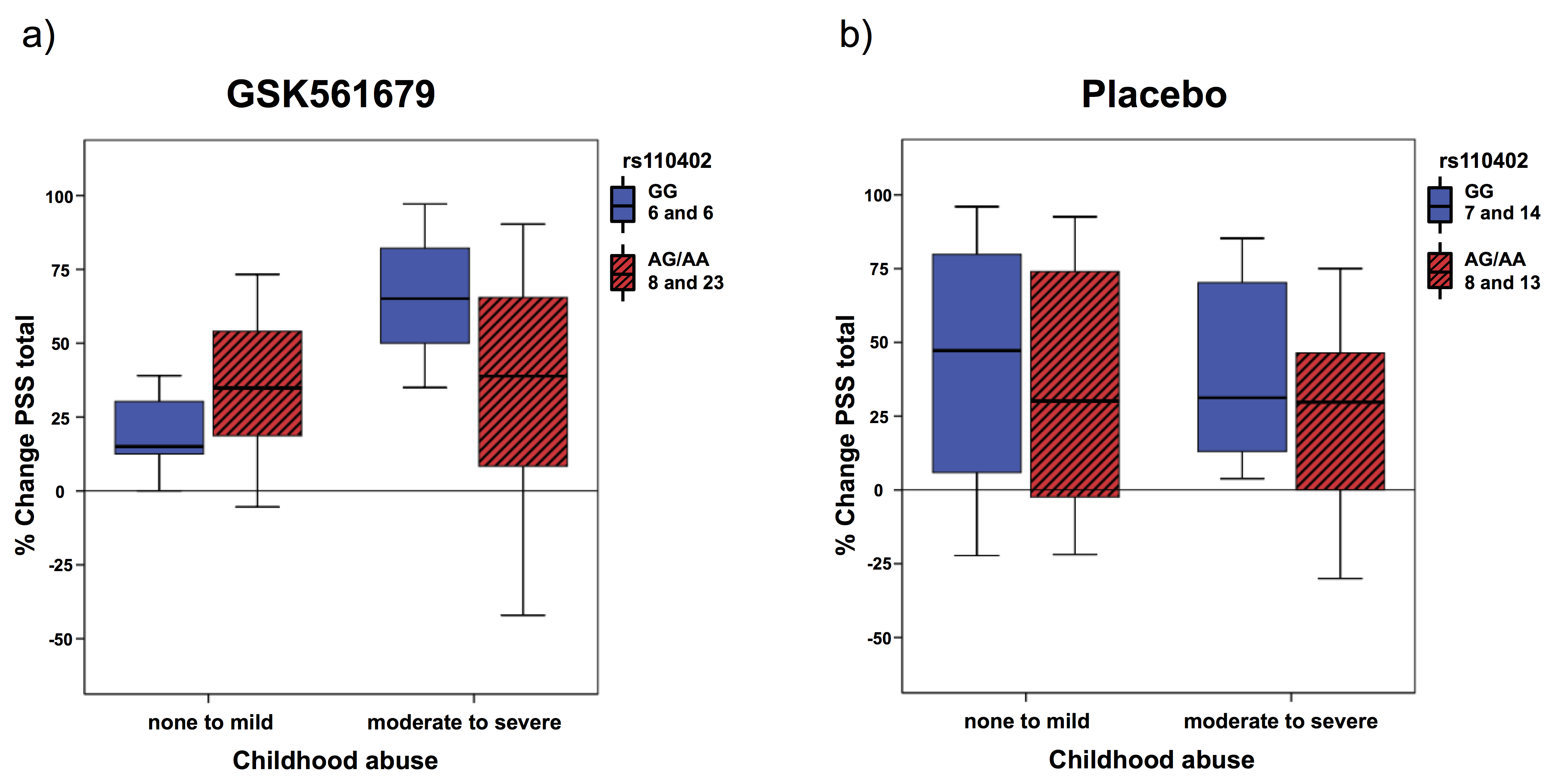
**

**Figure S1** The boxplots describe the mean % change of PSS total score in abused and non-abused patients treated with the CRHR1 antagonist or placebo. GG carriers are shown in blue (plain boxes) and AA/AG in red (striped boxes). rs110402 A carrier status by childhood abuse exposure showed a significant interaction effect on PSS score % change over treatment in subjects treated with the CRHR1 antagonist (n=43; F(1, 31)=4.42; p=0.043) **(a)** but not in subjects treated with placebo (n=42, p>0.05) **(b)**. rs110402 GG carriers exposed to child abuse displayed the highest % change of PSS symptoms following CRHR1 treatment. (From Biological Psychiatry; Dunlop et al., 2017)

**Table S**1. CRHR1: List of CpGs used for analysis

| **CpG** | **Chr.** | **Genomic location (hg19)** |
| --- | --- | --- |
| cg18090064 | 17 | 43716542 |
| cg04194664 | 17 | 43716618 |
| cg16228356 | 17 | 43848958 |
| cg08929103 | 17 | 43860356 |
| cg04856689 | 17 | 43862033 |
| cg24063856 | 17 | 43863304 |
| cg13947929 | 17 | 43863356 |
| cg27410679 | 17 | 43866279 |
| cg16642545 | 17 | 43878770 |
| cg00022871 | 17 | 43884359 |
| cg00025823 | 17 | 43909151 |

**Table S2**. NR3C1: List of CpGs used for analysis

| **CpG** | **Chr.** | **Genomic location (hg19)** |
| --- | --- | --- |
| cg17860381 | 5 | 142783570 |
| cg04111177 | 5 | 142783608 |
| cg15910486 | 5 | 142783621 |
| cg15645634 | 5 | 142783639 |
| cg18068240 | 5 | 142783844 |

**Table S3**. FKBP5: List of CpGs used for analysis

| **CpG** | **Chr.** | **Genomic location (hg19)** |
| --- | --- | --- |
| cg16012111 | 6 | 35656758 |
| cg07843056 | 6 | 35656848 |
| cg01294490 | 6 | 35656906 |
